# Extracellular vesicles mediate signaling between the aqueous humor producing and draining cells in the ocular system

**DOI:** 10.1371/journal.pone.0171153

**Published:** 2017-02-27

**Authors:** Natalie Lerner, Sofia Avissar, Elie Beit-Yannai

**Affiliations:** Clinical Biochemistry and Pharmacology Department, The Faculty of Health Sciences, Ben-Gurion University of the Negev, Beer-Sheva, Israel; National Cancer Center, JAPAN

## Abstract

**Purpose:**

Canonical Wnt signaling is associated with glaucoma pathogenesis and intraocular pressure (IOP) regulation. Our goal was to gain insight into the influence of non-pigmented ciliary epithelium (NPCE)-derived exosomes on Wnt signaling by trabecular meshwork (TM) cells. The potential impact of exosomes on Wnt signaling in the ocular drainage system remains poorly understood.

**Methods:**

Exosomes isolated from media collected from cultured NPCE cells by differential ultracentrifugation were characterized by dynamic light scattering (DLS), tunable resistive pulse sensing (TRPS), and nanoparticle tracking analysis (NTA), sucrose density gradient migration and transmission electron microscopy (TEM). The cellular target specificity of the NPCE-derived exosomes was investigated by confocal microscopy-based monitoring of the uptake of DiD-labeled exosomes over time, as compared to uptake by various cell lines. Changes in Wnt protein levels in TM cells induced by NPCE exosomes were evaluated by Western blot.

**Results:**

Exosomes derived from NPCE cells were purified and detected as small rounded 50–140 nm membrane vesicles, as defined by DLS, NTA, TRPS and TEM. Western blot analysis indicated that the nanovesicles were positive for classic exosome markers, including Tsg101 and Alix. Isolated nanoparticles were found in sucrose density fractions typical of exosomes (1.118–1.188 g/mL sucrose). Using confocal microscopy, we demonstrated time-dependent specific accumulation of the NPCE-derived exosomes in NTM cells. Other cell lines investigated hardly revealed any exosome uptake. We further showed that exosomes induced changes in Wnt signaling protein expression in the TM cells. Western blot analysis further revealed decreased phosphorylation of GKS3β and reduced β-catenin levels. Finally, we found that treatment of NTM cells with exosomes resulted in a greater than 2-fold decrease in the level of β-catenin in the cytosolic fraction. In contrast, no remarkable difference in the amount of β-catenin in the nuclear fraction was noted, relative to the control.

**Conclusions:**

The data suggest that NPCE cells release exosome-like vesicles and that these nanoparticles affect canonical Wnt signaling in TM cells. These findings may have therapeutic relevance since canonical Wnt pathway is involved in intra-ocular pressure regulation. Further understanding of NPCE-derived exosome-responsive signaling pathways may reveal new targets for pharmacological intervention within the drainage system as a target for glaucoma therapy.

## Introduction

### Glaucoma and the ocular drainage system

The privilege of age-related diseases is predicted to increase in the coming year and glaucoma patient number worldwide is expected reach 80 million by 2020, despite current advances in therapy[1].

Glaucoma is characterized by the ongoing deterioration of the retinal ganglion layer and worsening of visual field defects, accompanied changes in the optic nerve head. High intraocular pressure (IOP) has long been considered the most important risk factor for the onset and progression of glaucoma, and therefore pharmacological and surgical treatments have focused on lowering the IOP. However, even with treatment to lower IOP and even in normal tension glaucoma [2], optic nerve damage may progress.

The ocular drainage system comprises a number of unique tissues serving specialized functions. In glaucoma diseases, the delicate balance between the tissue producing the aqueous humor (AH), i.e., the ciliary epithelium, and the AH-draining tissues, i.e., the trabecular meshwork (TM) and Schlem's canal, is essential for maintenance of the intra-ocular pressure (IOP). Communication between these tissues was first suggested by Coca-Prados and colleagues [3]. Still, the details of the hypothesized communication remain to be defined. Active proteins [4] have been detected in the AH, supporting the idea of tissue communication within the drainage system. Among AH proteins, some were uniquely identified in the AH, while many others, such as cytokines [5], kinases [6], phosphatases [7], growth factors [8], are general participants of cellular communication. Further support for communication between the ocular drainage systems came from cell culture experiments where co-cultured non-pigmented ciliary epithelium (NPCE) and TM cells induced significant increases in the activity of some phosphatases and MMPs (matrix metalloproteinases), MMP-2 and MMP-9,) in TM cells [9]. Still, it remains mechanistically unclear how active molecule proteins and enzymes known to be located in the intracellular moiety, could function in the AH which is found in the extracellular milieu. The involvement of Extracellular vesicles (EV) offers one possible solution.

### EV and exosomes

Exosomes are 30–140 nm-diameter membrane-bound extracellular vesicles that are shed by various types of cells under both physiological and pathological conditions [10]. Exosomes are part of a larger group of vesicles known as EV. Biological extraction methods of exosomes cannot exclude the presence of some larger vesicles and the exosomes size distribution is relatively large, thus the term EV is preferred and used along the present research. Initially thought to be a mechanism for removing unneeded proteins, today it is accepted that EV play a role in various biological systems, by acting as clearing vesicles [11], contributing to immune system modulation [12], serving as pathological biomarkers [13] and acting in signal transmission [14]. Signal transmission by EV is achieved by facilitating the transfer of proteins, functional mRNAs and microRNAs and might contribute to subsequent protein expression in target cells [10]. Limited data, however, is available regarding the role of extracellular vesicles (EV) in ocular physiology and pathology conditions.

### Ocular drainage system EV

TM-derived EV present the protein profile characteristic of EV but also contain unique proteins, including the glaucoma-causing protein myocilin [15]. TM cells respond to environmental challenges by releasing MYOC myocilin -associated EV [16,17], with such release from the retinal pigment epithelium being controlled by myocilin [18]. Recently AH-extracted EV were analyzed for protein content by Stamer and colleagues [19]. However, NPCE-derived EVs has yet to be described, and as such, were addressed in the present study. Specifically, we propose that EV are the mediators of signaling transference between the NPCE and the TM.

### The Wnt signaling pathway

Among the many potential signaling pathways that might be involved in the drainage system function, the Wnt signaling pathway is a prime candidate, as it has already been shown to play a role in glaucoma diseases [20,21]. Moreover, it was suggested that myocillin is a modulator of Wnt signaling [22]. Mao et al. showed that the canonical Wnt signaling pathway exists in the TM and that the non-canonical Wnt signaling pathway does not [23]. In the absence of Wnt, cytoplasmic β-catenin is phosphorylated and forms a complex with axin, APC, GSK3, and CK1. Phosphorylated β-catenin is recognized by ubiquitin E3 ligase and undergoes proteosomal degradation. In the presence of Wnt ligand, a receptor complex forms between Fz, LRP5/6 and Dvl, leading to LRP5/6 phosphorylation and axin recruitment. This, in turn, disrupts axin-mediated phosphorylation/degradation of β-catenin, allowing β-catenin to accumulate in the nucleus, where it activates Wnt-responsive genes [24]. A recent report revealed the canonical Wnt signaling pathway to be an important modulator of extracellular matrix expression in the TM and provided a mechanistic framework for its regulation of outflow facility and IOP [20]. We suggest that the trabecular meshwork Wnt pathway can be modulated by NPCE derived EV affecting downstream the drainage system extracellular matrix stiffness and AH drainage resistance. Understanding the roles EV serve within the ocular drainage system could provide new vistas for pharmacological intervention.

## Materials and methods

### Cell lines

A human normal trabecular meshwork (NTM) cell line was generously donated by Alcon Laboratories, Fort Worth, TX, and maintained in Dulbecco's modified Eagle's medium (DMEM) containing 10% fetal calf serum (FCS), 2 mM L-glutamine, 100 μg/ml streptomycin and 100 units/ml penicillin (all from Biological Industries, Kibbutz Beit Ha-Emek, Israel) in a humidified atmosphere of 95% air and 5% CO_2_ at 37°C. Human non-pigmented ciliary epithelial (NPCE) and ciliary muscle (HCM) cell lines were kindly supplied by Prof. Miguel Coca-Prados, Yale University. NPCE and RPE cells were cultured in DMEM depleted of FCS-derived EV by overnight centrifugation at 100,000 g. HCM cells were propagated in DMEM supplemented with glutamine and antibiotics. Human ovarian carcinoma (SKOV-3), brain microvascular endothelial (cEND), breast cancer (MCF-7) and prostate adenocarcinoma (LANCaP) cell lines were kindly provided by Dr. Ayelet David, Ben-Gurion University. cEND and MCF-7 cells were grown in DMEM, while SKOV-3 and LNCaP cells were grown in RPMI (Biological Industries, Kibbutz Beit Ha-Emek, Israel). Both media were supplemented with 10% FCS, 2 mM L-glutamine, 100 μg/ml streptomycin and 100 units/ml penicillin in a humidified 5% CO_2_ atmosphere. A human retinal pigment epithelium (RPE) cell line was a gift from Dr. Zeev Dvashi, Kaplan Medical Center, Rehovot, Israel, and cultivated under standard conditions in a 1:1 mixture of DMEM and Ham's F12 nutrient mixture (DMEM/F12) containing FCS and antibiotics.

### Exosome extraction

Exosome purification was performed according to a protocol adapted from Thery et al. [25]. Briefly, EV were extracted from the supernatant of NPCE cells and cultured for 48 h in medium containing exosome-depleted FCS. Cell culture supernatants were collected and subjected to serial centrifugations, namely 300 g for 10 min, 2,000 g for 10 min, 10,000 g for 30 min, all at 4°C. EV were then pelleted at 100,000 g for 70 min and washed with PBS and recovered at 100,000 g for 70 min. The pelleted EV were re-suspended in PBS and the quantity of exosomal protein was measured by Bradford assay [26] (Bio-Rad).

### Sucrose density gradients

For sucrose density gradient analysis [25] of EV, the vesicle-containing pellet was re-suspended in 2 mL HEPES/sucrose (2.5 M sucrose in 20 mM HEPES) and loaded onto a continuous sucrose gradient (0.25–2.0 M in 20 mM HEPES, pH 7.2) prepared using a Hoefer SG30 gradient maker. The gradients were centrifuged overnight at 210,000 g at 4°C in a SW-41 swinging bucket rotor. One ml fractions were collected from the top to bottom of each gradient and 50 μl aliquots were used for density determination. The refractive index of each fraction was measured at 22°C using an Abbe refractometer (Atago 1211 NAR-1T liquid) and converted to density using the conversion table published in the Beckman Coulter ultracentrifugation manual, as described previously [25]. Next, each fraction was washed in 20 mM HEPES, ultra-centrifuged at 100,000 g for 1 h and mixed with lysis buffer prior to analysis by Western blotting.

### Western blot analysis

Western immunoblotting of sucrose density gradient fractions was performed on 10% polyacrylamide gels to confirm the presence of specific exosomal marker proteins. Pellets obtained from sucrose density gradient fraction were re-suspended in lysis buffer (20 mM Hepes, 150 mM NaCl, 1 mM EGTA, 1 mM EDTA, 10% glycerol, 1 mM MgCl_2_, 25 mM NaF, 20 μl/ml protease inhibitor cocktail) and sonicated for 10 s at 4°C. Protein content was measured according to the Bradford method [26]. All fractions were diluted (1:3) in loading buffer (10% (v/v) glycerol, 20% (v/v) SDS, 5% (v/v) β-mercaptoethanol, and 0.05% (w/v) bromophenol blue) and heated for 5 min at 95°C. Samples were separated on SDS-PAGE gels and transferred to nitrocellulose membranes using an electro blotting apparatus. After blocking in TBST (Tris Buffer Saline + Tween) buffer containing 5% milk for 1 h at room temperature, the membrane was probed with primary antibodies against Alix (ABC40 rabbit polyclonal antibodies, Millipore; 1:5,000 dilution) or Tsg101 (GTX70255 mouse monoclonal antibodies, GeneTex; 1:800 dilution). Membranes were then washed and incubated with secondary HRP-conjugated antibodies. Secondary antibody binding was visualized with an enhanced chemiluminescence detection (ECL) kit (Amersham Pharmacia Biotech). In addition, Western Blot analysis was performed on protein lysates from NTM cells either untreated (Ctrl) or treated at various time points (0.5, 1, 2, 4, 6, 8 and 24 h) with NPCE-derived EV (20 μg/1.5x10^7^ cells). NTM cells were cultured on 24 well plates and pre-incubated in absence or presence of EV. The cells were washed with ice-cold PBS and lysed with lysis buffer. Proteins were transferred to nitrocellulose membranes and probed with primary antibodies against phospho-GSK3β (5B3 rabbit monoclonal antibodies, Cell Signaling; 1:000 dilution), β-catenin (D10A8 rabbit monoclonal antibodies, Cell Signaling; 1:1000 dilution) and GAPDH (sc-25778 rabbit polyclonal antibodies, Santa Cruz; 1:400 dilution). The membranes were then incubated with secondary antibodies (GE Health Life Sciences Na934v anti-rabbit or Jackson Immunoresearch Laboratories 112035062 anti-mouse antibodies). Immune-complexes were detected with ECL reagent, followed by exposure to Kodak X-ray film. The films were scanned, and densitometry was performed using by EZQuant software (EZQuant Biology Solution).

### Exosome antibody arrays

The Exo-Check antibody array (System Biosciences, Mountain View, CA) was used to detect known exosome markers. Briefly, exosome protein lysates were prepared by adding 600 μl of exosome lysis buffer to 250 μg of exosome protein. The antibody membrane array was placed in distilled water at room temperature for 2 min. Exosome lysate/binding mixture was added to the antibody membrane and then incubated overnight on a shaker at 4 C. After washing with array wash buffer, detection buffer was added to the membrane and incubated at room temperature for 2 h. The membrane was washed twice with wash buffer and then the immune-complexes were detected with ECL reagent, followed by exposure to Kodak X-ray film for the final signal analysis.

### Coomassie brilliant blue staining

EV were extracted as described above. Isolated EV were sonicated, solubilized with loading buffer and separated by 10% SDS-PAGE. The gels were then stained in the Coomassie brilliant blue solution (0.1% Coomassie blue in 10% acetic acid, 50% methanol) and shaken at room temperature for 1 h. The gels were destained by soaking for 2 h in destaining solution (10% acetic acid, 50% methanol) and dried between sheets of cellophane (Gel drying frames, Sigma).

### Real-time PCR analysis

NTM cells were seeded at density of 1x10^6^ cells/well in 6-well plates. After 48h, cells were exposed for 1, 2, 4, 6 and 24h, to NPCE EV 2 μg/mL, or DMEM alone as control. Total RNA was isolated using an EZ-RNA Kit (Biological Industries) according to the manufacturer’s instructions. RNA quality and quantity were assessed at 260 nm using a NanoDrop2000 Spectrophotometer (Thermo Scientific). Equal amounts (1μg) of RNA were reverse-transcribed in triplicates using qScript cDNA Synthesis kit (Applied Biosystems). Changes in mRNA levels for Wnt target genes *Axin 2* and *LIF1* were determined by real-time PCR with an Applied Biosystems Real Time PCR 7500 system (Applied Biosystems) using TaqMan Fast Advanced Master Mix according to the manufacturer’s instructions (Applied Biosystems). The incubation and thermal cycling conditions were as follows: hold for 20 s at 95°C, denaturation for 3 s at 95°C, annealing and extension for 30 s at 60°C. The number of cycles was 40. The threshold cycle was measured as the cycle number at which sample fluorescence increases statistically above background and crossing points for each transcript. Human GAPDH gene was used as endogenous control. The mRNA levels were calculated using 7500 Software v 2.0.4 (Applied Biosystems).

Transmission electron microscopy at cryogenic temperatures (cryo-TEM). For cryo-TEM, a 4 μl aliquot of the vesicle suspension was applied to a copper grid coated with perforated lacy carbon 300 meshes (Ted Pella) and blotted with filter paper to obtain a thin liquid film of solution. The blotted sample was immediately plunged into liquid ethane at its freezing point (-183°C) with an automatic plunger (Lieca EM GP). The vitrified specimens were transferred into liquid nitrogen for storage. Sample analysis was carried out with a FEI Tecnai 12 G2 TEM at 120 kV with a Gatan cryo-holder maintained at -180°C. Images were recorded with the Digital Micrograph software package at low dose conditions to minimize electron beam radiation damage.

### Tunable Resistive Pulse Sensing (TRPS)

Exosomal samples were passed through 0.22 μm filters to eliminate contaminating debris. TRPS measurements were performed using a qNano (qIzon Science, Christchurch, New Zealand) instrument equipped with aNP150A type membrane. The apparatus was operated at a voltage of 0.64 V and a pressure equivalent to 7 cm of H_2_O. The membrane was stretched to 46.26 mm. Polystyrene beads at a concentration of 1.x10^10^ beads/mL (11o nm; Izon Science) were used to calibrate size and concentration, following the manufacturer’s instructions. Samples were diluted 500-fold with PBS buffer and measured over 10 minutes.

### Nanoparticle Tracking Analysis (NTA)

The concentration and size distribution of the EV were measured with a NanoSight NS500 instrument (Malvern Instruments, Ranch Cucamonga, CA). Briefly, the exosomal fraction was diluted 1:1000 with PBS at 23°C and size dispersion was measured. A video 30–60 s in duration was taken with a frame rate of 30 frames /second, and particle movement was analyzed by NTA software (version 2.2, NanoSight). NTA (Nanoparticle tracking analysis) post-acquisition settings were optimized and kept constant between samples. Each video was analyzed for mean, mode, and median vesicle sizes, together with an estimate of vesicle concentration.

### Extracellular vesicles labeling and confocal tracing

Extracellular vesicles-containing pellets were re-suspended in 1 mL of PBS containing 5μg/ml DiD(1,1′-dioctadecyl-3,3,3′,3′-tetramethyl-indodi- carbocyanine,4-chloro-benzenesulfonate salt Biotium, Hayward, CA) and incubated for 10 min. After ultracentrifugation at 100,000 g for 70 min at 4°C, the exosome-containing pellet was washed in PBS and centrifuged again for 90 min at 150,000 g to remove unincorporated DiD. To assess the kinetics of exosome internalization, labeled EV were re-suspended in DMEM supplemented with 10% vesicle-free FCS. DMEM-re-suspended vesicles were added to NTM cells (20 μg per 1.5x10^7^ cells) for various periods of time (0.5, 1, 2, 4, 8, 24 and 32 h). The cells were washed with PBS, fixed with 4% paraformaldehyde, permeabilized with 0.1% Triton X-100 and stained with α-tubulin antibodies (62901 mouse polyclonal antibodies, Biolegend; 1:200 dilutions). Cells were then washed and Alexa Fluor 488-conjugated anti-mouse IgG (Jackson Immunoresearch Laboratories) were added as secondary antibodies for 1 h in the dark at room temperature. Samples were washed and mounted on slides in Fluromount-G mounting media (Southern Biotech). Images were analyzed with a FV1000-IX81 confocal microscope (Olympus, Tokyo, Japan) equipped with 60X objective. Pictured slides (30min / cell type) were analyzed for DiD labeled NPCE derived EV by Image J software

### NPCE exosome targeting specificity

Seven different human cell lines (NTM, HCM, RPE, LNCaP, SKOV-3, CEND and MCF7) were seeded on cover glasses in 24-well plate (70,000 cells/well) in growth medium complemented with exosome-depleted FCS on cover glasses (Marienfeld). Twenty-four h later, the medium was replaced with medium containing NPCE-derived DiD-labeled EV (2 μg DiD stain/well). Following 12 h incubation, the medium was removed, the cells were fixed with 4% paraformaldehyde, stained with DAPI and α-tubulin antibodies and examined by confocal microscopy.

### Flow cytometry

NTM cells were seeded in 6-well plates at a density of 1x10^6^ per well in 3 mL of the appropriate medium. After cell adhesion, the cells were cultured with NPCE-derived EV (20 μg per 1.5x10^7^ cells) for 24 h. The cells were washed twice with cold PBS and detached by trypsinization, followed by centrifugation and resuspension in PBS. The samples were fixed with 80% methanol for 5 min and permeabilized with 0.1% PBS-Tween for 20 min. For immunostaining, the cells were blocked by incubation with 3% BSA in PBS on ice for 30 min. Intracellular staining was performed by adding mouse anti-pan cadherin antibodies (ab6528, 2 μg/1x10^6^ cells) for 30 min at 22°C. Subsequently, the samples were washed and incubated with Alexa-488-conjugated anti-mouse antibodies at a dilution 1:500 in the dark for 30 min at 22°C. After washing, the stained cells were re-suspended in PBS and fluorescence intensity was determined in a flow cytometer (Guava Easy Cyte mini, Guava Technologies).

### Statistics

Data are presented as mean ± standard deviation. Statistical evaluation of one way ANOVA was performed with GraphPad Prism version 5.0 software. Differences between groups were tested using Tukey's test.

The analysis of NTM cell treatment with EV along various time points was performed using SPSS (IBM SPSS Statistics, Version 23.0. Armonk, NY: IBM Corp). The analysis was performed on the mean values of two technical observations. All tests were considered significant at P ≤ 0.05. Parametric tests were conducted as data fitted the relevant assumptions of normality and homogeneity of variance. Two Way Analysis of Variance with NPCE or RPE derived EV treatments, NTM EV exposure time and their reciprocal interactions were applied.

## Results

### Characterization of EV isolated from NPCE cell culture supernatant

**Fig 1:**
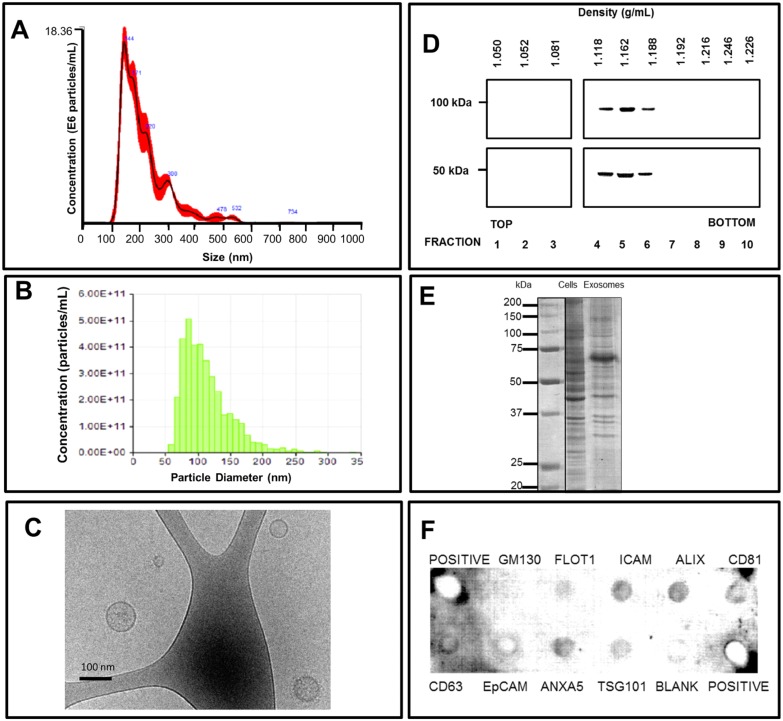
Characterization of exosomes isolated from NPCE cell culture supernatants. Size distribution of NPCE cell-derived exosomes as measured by (A) nanoparticle tracking analysis, (B) and tunable resistive pulse sensing (TPRS). (C) Cyto-TEM image of purified NPCE-derived exosomes. Scale bar: 100 nm. (D) Western blot analysis of exosomes floated on a continuous sucrose density gradient. 20 μg of exosomal proteins were loaded onto a continuous sucrose density gradient. Fractions collected from the gradient were separated by SDS-PAGE and probed for the exosomes markers Alix and TSG101. (E) Coomassie brilliant blue–stained SDS polyacrylamide gel after separation of 15 μg of total cell lysates or exosomal proteins from NPCE cells. (F) Exosomes antibody array marker analysis with Exo-Check, representative results.

EV extracted from NPCE were characterized by several analytic approaches. Exosome size was measured using a spectral method based on NTA (Fig 1A), TRPS (Fig 1B), while surface potential was determined using TRPS technology. [27]. Slight differences in exosome size were obtained by each method, namely 127.8±7.5, and 114±2.95 nm for NTA, and TRPS, respectively. The typical round structure of the EV was detected by cyo-TEM. The characteristically round EV were surrounded by a lipid bilayer, with a mean diameter 99.88±7.40 nm, yet ranging from 36 to 174 nm (Fig 1C). Exosomes can be characterized according to their density and accordingly were reported to have a density of 1.15 to 1.19 g/ml (Fig 1D). [25,28]. Upon refractometer analysis of the separated sucrose density gradient, the NPCE-derived exosome-containing layers were isolated and the EV were purified by ultracentrifugation. Such analysis revealed the NPCE-derived EV at a sucrose density of 1.118–1.188g/mL. Many EV were reported to contain proteins that are part of the exosome biogenesis machinery. These included Tsg101, a component of the endosomal sorting complex required for transport (ESCRT)-I, and Alix, a protein involved in ESCRT-II connections. Both proteins are recognized as markers for exosomal identification [29,30,31] (Fig 1D). The NPCE-derived EV also contained a large number of proteins. Coomassie brilliant blue staining of these proteins, separated by SDS-PAGE under reducing conditions, revealed a distinct profile that included multiple unique protein bands not seen in the NPCE cell lysate (Fig 1E).

Finally, using Exo-Check kit three different EV categories- trans-membrane or lipid bound extracellular proteins, cytosolic proteins and intracellular proteins were found to be positive (Fig 1F).

### Uptake of NPCE-secreted EV by NTM recipient cultured cells

**Fig 2:**
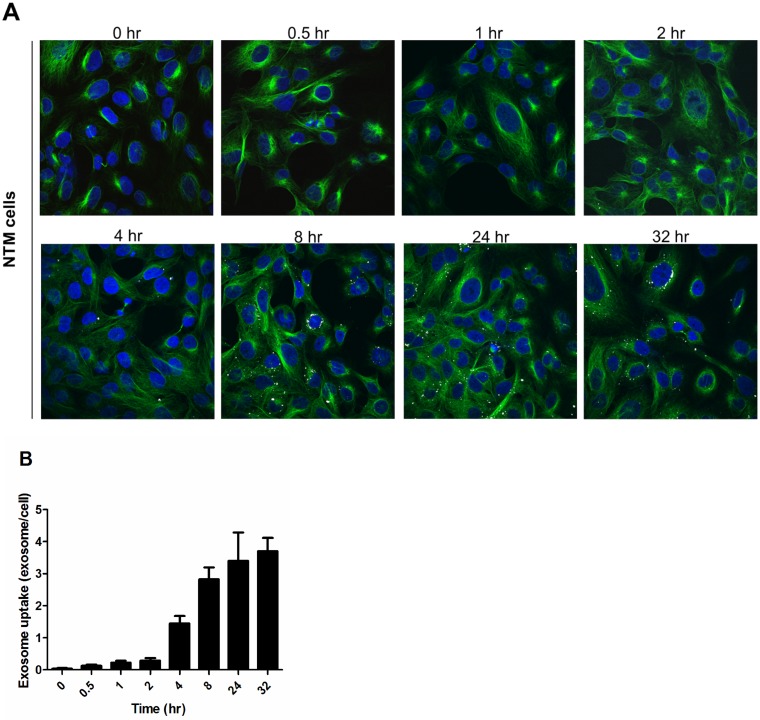
Uptake of NPCE-secreted exosomes by NTM recipient cultured cells within 32 h. (A) Representative confocal images of NPCE-derived exosomes internalized by NTM cells. Purified exosomes were labeled with the dye DiD (white) and added to cells of the NTM line (20 μg per 1.5x10^7^cells). After incubation with exosomes for the indicated times, the cells were stained with an α-tubulin monoclonal antibody (green), DAPI (blue) and visualized by confocal microscopy. (B) The graph presents exosome dynamics by spot count of internalized labeled exosomes. Data are average ± SD of three independent experiments. A minimum of 350 cells were analyzed for each sample. *P<0.05, **P<0.01, ***P<0.001.

Under physiological conditions, NPCE-derived EV are assumed to be released to the ocular anterior and posterior chambers [19]. The existence of EV in human aqueous humor was also recently reported [19]. To test our hypothesis that EV are the mediators of signaling transference between the NPCE and the TM, we examined the kinetics of exosome accumulation in a TM cell culture over time. A known amount of DiD-labeled NPCE-derived EV was incubated with TM cells and traced using confocal microscopy. Representative microscopic fields were counted for labeled EV. A continuous increase in accumulated exosome levels in the TM cells was found to be significant at 4 h. A maximal amount was reached by 12 h. This level persisted for up to 32 h more. The EV were found in the TM cytosol, being mainly localized in a perinuclear region (Fig 2A and 2B).

### Specificity of the cellular uptake of NPCE-derived EV by TM cells

**Fig 3:**
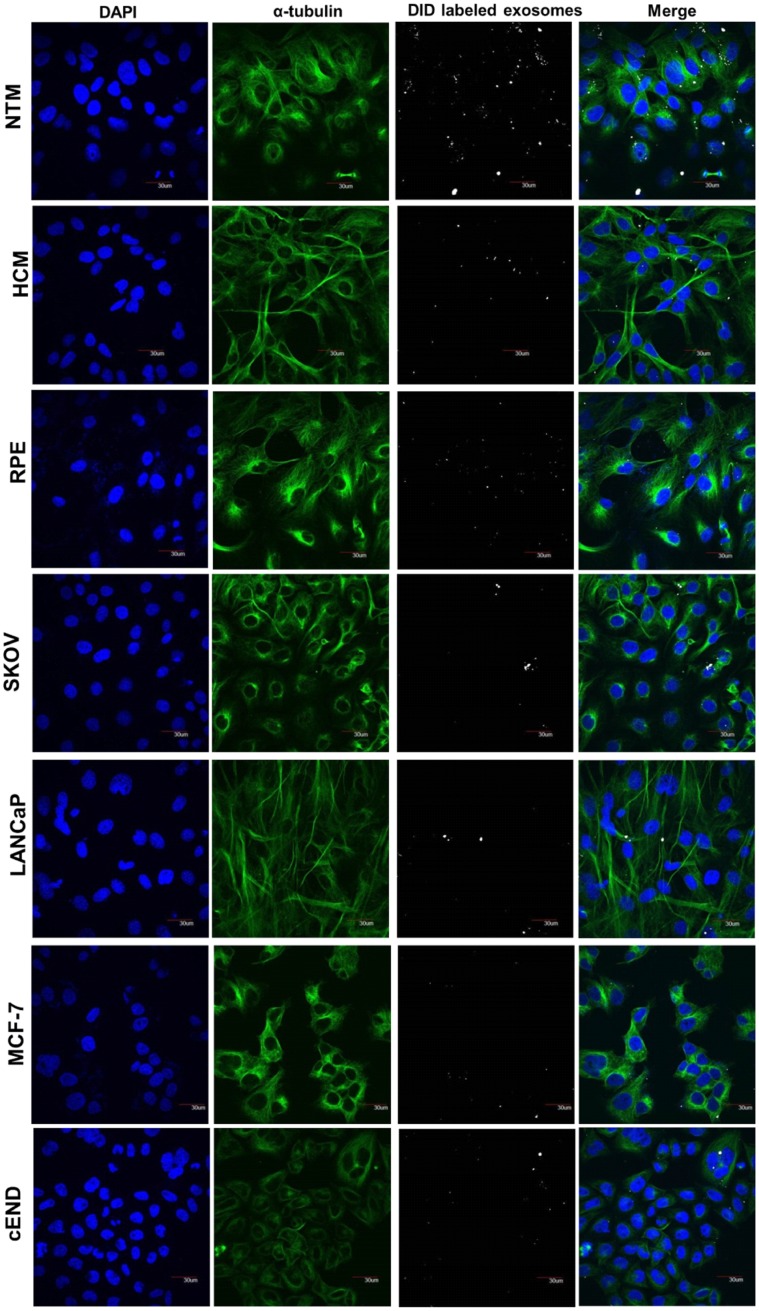
Confocal microscopy of cellular uptake of DiD-labeled exosomes by different cell lines. DiD-labeled (white) NPCE-derived exosomes were incubated for 12 h with NTM, HCM, RPE, SKOV-3, LANCaP, CEND and MCF-7 cells. After incubation, the cells were washed, fixed and labeled using Alexa Fluor 488-labeled anti-α-tubulin antibodies (green) to mark the cytoskeleton and mounted in mounting medium containing DAPI (blue). Images were acquired using a confocal microscope. Scale bar: 30 μm.

**Fig 4:**
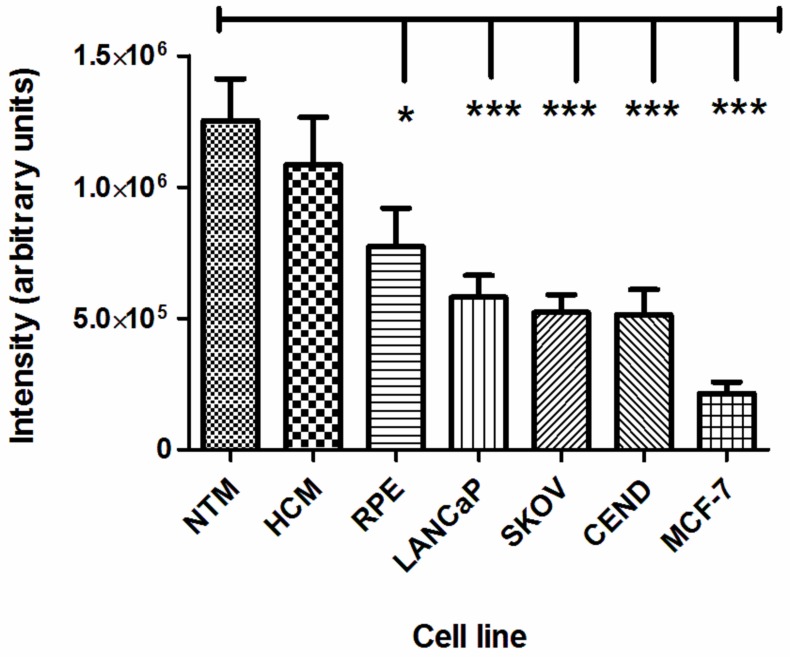
Quantitative cellular uptake of DiD-labeled exosomes by different cell lines. Fluorescence intensity analysis of DiD-labeled NPCE-derived exosomes entry into each cell type after a 12 h incubation, by Easy-Quant. *P<0.05, **P<0.01, ***P<0.001.

The mechanism behind the uptake of NPCE-derived EV by TM cells is unknown. Non-specific diffusion or uptake, as opposed to specific exosome recognition by a membrane transporter, could account for EV entering target cells [32]. One suggested pathway involves free diffusion followed by docking and then internalization [33]. In the present study, we compared the ability of different cell lines to accumulate DiD-labeled NPCE-derived EV. These included ocular (HCM, RPE), epithelial (MCF7, SKOV-3, LnCap), and endothelial (CEND) cells. All of the different cell types were grown to the same density and then incubated with the DiD-labeled NPCE-derived EV for 12 h, at which point exosome accumulation was verified. EV could be easily identified in those TM cells incubated with NPCE. In all of the other cell lines investigated, except HCM cell line, only very few NPCE-derived EV could be detected (Figs 3 and 4).

### Effect of NPCE-derived exosome treatment on the Wnt signaling pathway of NTM cells

**Fig 5:**
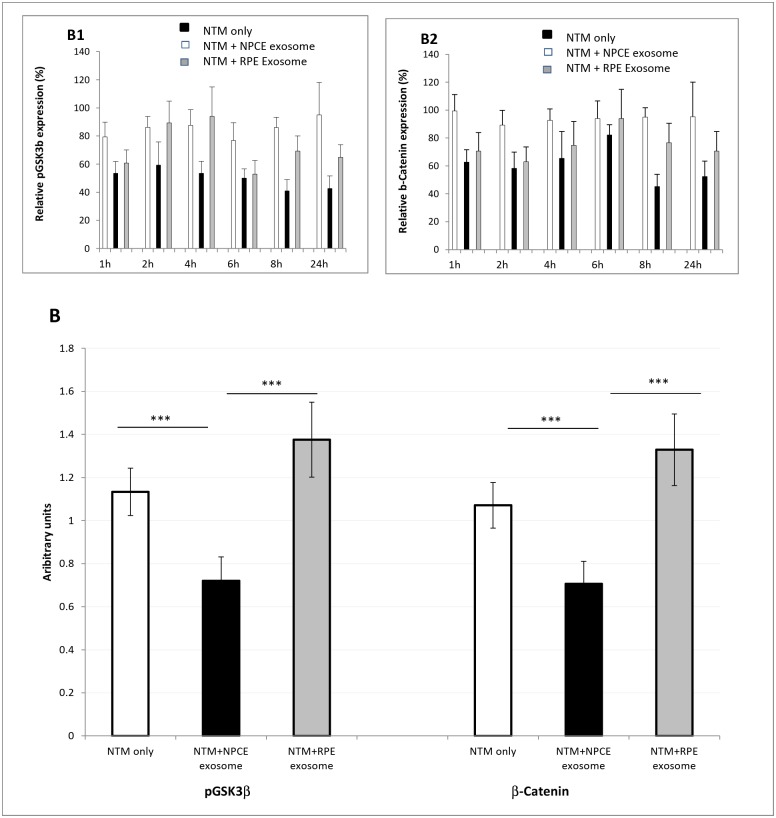
Effect of NPCE-derived exosome treatment on the Wnt signaling pathway in NTM cells. Lysates from NTM cells untreated or exposed to NPCE-derived exosomes or RPE derived exosomes were subjected to immunoblot analysis to detect cytosolic β-catenin or phospho-GSK3β. β-actin was used as a loading control. Data represent means ± S.D from minimum three independent experiments. (A) A representative Western blot showing expression of β-catenin (A1) and phospho-GSK3β (A2) at various time points. (B) Histograms presenting mean interaction of the phospho-GSK3β and β-catenin expression. ***P<0.001. (B1 & B2 inserts) A time and treatment histogram chart of the relative expression of β-catenin and phospho-GSK3β.

In the absence of Wnt, cytoplasmic β-catenin forms a complex with axin, APC, GSK3, and CK1, and is phosphorylated by CK1 and subsequently, by GSK3. Phosphorylated β-catenin is recognized by a specific ubiquitin ligase, which targets β-catenin for proteosomal degradation [24]. In the presence of Wnt ligand, a receptor complex forms between Frizzled and LRP5/6. Dvl recruitment by Frizzled leads to LRP5/6 phosphorylation and axin recruitment. This disrupts axin-mediated phosphorylation of β-catenin, allowing β-catenin to translocate to the nucleus where it can drive Wnt-dependent gene expression, bind to cadherin-mediated cell adhesions to modulate actin coupling, or be degraded following ubiquitination [24]. Changes in Wnt protein levels in NTM cells induced by NPCE-derived EV were evaluated by Western blot analysis. We used two way ANOVA analyses in order to study the possible influence of treatment and length of exosome exposure mutual effects. Phosphorylated GSK3β mean treatment (EV derived from NPCE, RPE or control) was significantly different (p≤0.003) while interaction (p≤0.978) and exosome exposure length (p≤0.959) were not. For pGSKβ3, the control non-treated NTM cells (1.13±0.11) and the NTM cells treated with RPE derived exosome (1.38±0.17) were significantly higher than NPEC derived EV treated NTM cells (0.72±0.11), but did not differ significantly from each other. β-Catenin mean exosome exposure length (p≤0.002) and treatment (p≤0.001) were highly significant, while interactions (p≤0.672) were not significant. For β-Catenin, the control non-treated NTM cells (1.07±0.11) and the NTM cells treated with RPE derived exosome (1.33±0.17) were significantly higher than NPEC derived EV treated NTM cells (0.71±0.11), but did not differ significantly from each other (Fig 5B). Decreased phosphorylation of GKS3β and decrease in β-catenin cytosolic levels were detected in NPCE derived exosome treatment along the different time points while no reduction was found for the RPE derived exosome treatment (Fig 5C).

### Effect of NPCE-derived exosome treatment on the selected mRNA expression in NTM cells

**Fig 6:**
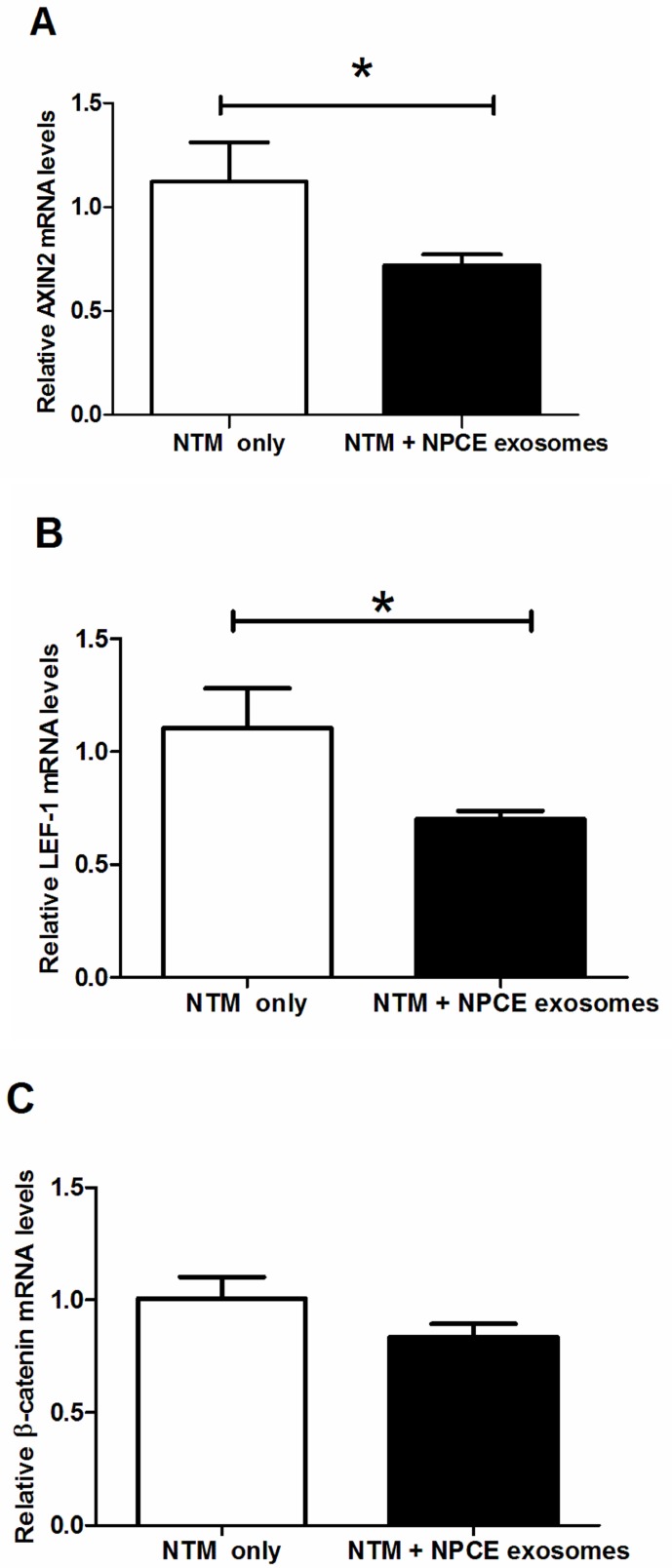
Real-time qRT-PCR was performed to examine the mRNA expression levels (mean ± sem) of canonical WNT/β-CATENIN signaling components, including the Wnt transducers Axin2 (A), β-catenin (C), and the Wnt transcription factors LEF1 (B). Data were normalized to the average mRNA level of GADPH. Statistical difference is indicated by asterisks (*P < 0.05,).

Endogenous expression of Wnt-regulated gene following NPCE derived exosome exposure was analyzed for possible up-regulation at various time points. *AXIN2* and *Lif1* genes are often upregulated by the Wnt signaling pathway and used as a marker for canonical Wnt/ β-catenin pathway activation [33,34]. In the present study we examine the expression of *beta catenin*, *AXIN2* and *Lif1* genes expression. qPCR studies revealed that treatment of NTM cells with NPCE derived EV for two hours significantly reduced *AXIN2* and *Lif1* expression vs. their untreated controls (Fig 5A & 5B, *P* < 0.05, *n* = 6). Beta catenin mRNA levels showed a trend of mRNA reduction that did not reach significance (Fig 6C). At all the other time points of NTM cells exposed to NPCE derived EV, no significant changes in mRNA levels were found versus untreated NTM cells.

### Effect of EV on the expression of cell-to-cell adhesion proteins

**Fig 7:**
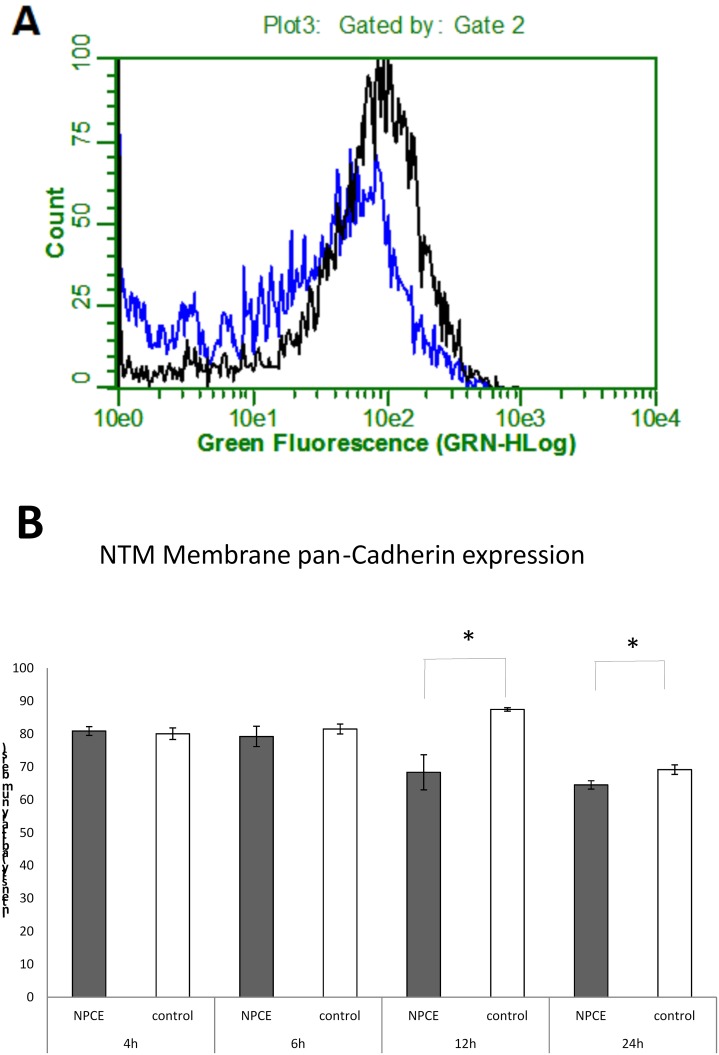
Flow cytometry analysis of the expression of pan-cadherin in the presence or absence of NPCE-derived exosomes. Isolated exosomes were added to NTM cells for 4, 6,12 and 24 h. Cells were collected permiablized and stained with monoclonal antibodies against the intracellular membrane domain of pan-cadherin. Acquisition of >5,000 events was performed. A representative experiment is shown in A. Results (mean ± SE) of three independent experiments are shown in B. *P<0.05, **P<0.01.

To elucidate whether EV induce changes in adhesion protein levels, we studied the expression of membrane cadherin, cell-cell adhesion proteins, by Western blot following 24h incubation with NPCE derived EV and flow cytometry following 4, 8,12 and 24h incubation with NPCE derived EV. The selected antibody used was capable of detecting the intracellular domain of the membrane anchored cadherins. Western blot analysis of NPCE-derived EV induced a significant (p<0.01) decrease in pan-cadherin expression, suggesting a link between the NPCE derived EV and the trabecular meshwork adhesion molecules (Fig 7).

## Discussion

Aqueous humor, previously considered as a nutrient supplier and waste product disposal from the ocular front section [35], is now accepted as a mediator of cell-cell communication. Proteomic analysis demonstrated that hundreds of protein are present in AH [36] and that their composition changes under pathological conditions [37]. The presence of active factors in the ocular drainage system suggests that they assume an active role in drainage system homeostasis and pathology. Still, which tissue releases these active factors and what is their specific target remain open questions [6,7,19].

Cell lines were used in the present study in order to explore the basic existence of the EV mediated signaling within the ocular drainage system. Limited data is available regarding the in-vivo human aqueous humor extracellular vesicles concentrations and large differences were reported, 8.6 to 62.3 × 10^8^ particles/ml in a single report by Dismuke at al. [19]. Moreover, EV concentration is effected by the extraction procedure. The ratio between EV and NTM cells was 1 to 100 similarly to another EV mediated signaling research [38]. Research performed with cell lines has limitations such as mutations that can't fully reflect the physiology and pathology of normal primary cells. In addition there is no evidence that these exosomes are representative of exosomes found in aqueous humor or released in the eye by the non-pigmented ciliary epithelia. However we hypothesize that the present NPCE derived EVs first research opens a window to further research in EV mediated signaling within the drainage system using human ciliary epithelium and trabecular meshwork primary cutlers.

In the present study, we considered the idea that EV form at least part of the ocular drainage tissue communication process. Growth factors, cytokines [39], redox-active molecules [40], lipids [41] and nucleotide mobility are involved in exosome-mediated signaling [42]. The characterization of NPCE-derived EV conducted here demonstrated the presence of EV presenting a classic appearance in terms of shape and size, behavior on sucrose density gradients and content. The present study further used dye-labeled EV as a tool for tracing EV by confocal microscopy [43], as well as Western blot analysis and flow cytometry to measure changes in protein expression and activity.

We show here for the first time that NPCE-derived EV are carriers of active factors delivered to TM tissue where they interfere with the Wnt signaling pathway, as reflected by the alterations in both GKS3β and β-catenin levels, thus supporting previous reports that Wnt signaling plays a major role in the TM tissue drainage resistance via adhesion molecule changes and extracellular matrix remodeling [20,21,23]. RPE derived exosome were chosen as positive control following the report that exosome derived RPE cells are present in the aqueous humor [1]. This effect was found to be unique to the NPCE derived EV and not a general EV effect on NTM cells as RPE-derived exosomes did not cause a reduction in the tested Wnt protein expressions.

Regarding the drainage system, it was shown that TGF-β2 enhances cadherin-mediated cell–cell adhesion and β-catenin expression in human trabecular meshwork cells [44]. Wnt antagonist treatment resulted in long-term increases in human trabecular meshwork cell stiffness, accompanied by decreased β-catenin levels and increased phosphorylation [45]. Downstream of Wnt signaling, a series of cadherin proteins were shown to be involved in cell adhesion, as was the fact that this pathway is influenced by kinases and phosphatases activity [46]. As for the ocular drainage system, VE-cadherin, E-cadherin and N-cadherin were detected therein, although pan-cadherin detection was used in other studies. In the present efforts, by using an antibody capable of detecting the intracellular part of the different cadherins, a small but significant decrease in pan-cadherin expression was detected. As N-cadherin was reported to be abounded in the ocular drainage system [36], we assume that measuring a specific cadherin instead of total cytosolic cadherin might result in a larger difference in cadherin expression between exosome-treated and non-treated TM cells. The data suggest that NPCE-derived EV may decrease cadherin-mediated cell-cell adhesion. Changes in cell-cell adhesion could alter TM biomechanics and thus alter TM outflow by enhancing the aqueous humor outflow facility. Moreover, cadherins are membrane proteins. Our data was collected from whole TM cells, reflecting the total of cytosol, nucleus and membrane cadherins. Indeed, analysis of cadherin changes in the membrane alone might increase the differences in expression following exosome treatment seen.

The contribution of EV to signaling between neighboring tissues and even between relatively distant tissues during tissue development, cancer research, and upon activation of the immune and other systems was demonstrated [47]. In the limited numbers of papers on EV derived from human primary TM cells [15,16] and aqueous humor [19,48], the main focus was on the involvement of EV as myocilin carriers or stimulants of myocilin release. To the best of our knowledge, the use of EV as signal carriers between the two main tissues involved in aqueous humor production and release has not yet been previously addressed.

Several hypotheses can describe the mechanism of interaction between EV and cells. EV can deliver their signaling cargo in different manners, for instance, in a juxtacrine fashion [2]. Here, exosomal membrane proteins interact with receptors in a target cell and activate intracellular signaling. Alternatively, proteases present in the extracellular space could cleave exosome membrane proteins, with the freed proteins then acting as soluble ligands that could bind to target cell surface receptors [2]. This would, in turn, activate the signaling cascade within the target cell. Finally, exosome uptake by fusion [33] or endocytosis [43] with the target cell membrane and subsequent release of exosome contents into the recipient target cell in a non-selective manner is also conceivable. When incubating NPCE-derived EV with NTM cells in the current study, the data collected suggest that a significant number of EV were internalized by the NTM target cells. However in the present research the NPCE derived EV entry to the NTM cells is taking place in a longer pace than the Wnt signaling effects detected. This suggests that processing in a juxtacrine-like fashion and/or protease-based cleavage of NPCE-derived EV occurs simultaneously. The involvement of these two signaling delivery pathways thus requires further investigation.

The kinetics of NPCE-derived exosome internalization by NTM cells suggests that about four hours are needed for a significant exosome entry to have occurred. The maximum exosome level was found after 12 h and was maintained for up to 32 h. In a research conducted by Zhongdang Xiao and colleagues using live wide-field fluorescence and Z-stack confocal microscopy of PC12-derived EV, linear accumulation was observed for up to three hours of incubation [38]. Explanations that can account for the differences with our findings include those based on differences in the EV sources used, cell targets, duration of the experiments and tracing methods used.

Whether EV contains specific targeting molecules *in situ* that favor a particular tissue or cell type is an interesting and important question. In several studies, EV were shown to specifically target recipient cells where they would exchange proteins and lipids or to trigger downstream signaling events and deliver specific nucleic acid cargo [10,49]. Furthermore, the recent use of EV as engineered nucleic acid carriers for different diseases was suggested. However, this promising delivery method requires over-expression of specific proteins on the exosome surface so as to achieve reasonable cargo delivery [50,51].

The canonical Wnt signaling route plays a significant role in ocular drainage system homeostasis [23]. Myocilin induced stress fiber formation in human primary TM cells that could be blocked by inhibitors of Wnt signaling, secreted Frizzled-related protein 1 (sFRP1) or sFRP3 [22]. Yue et al. suggested that myocilin up-regulation induced Wnt activation and downstream cAMP/PKA activation and RhoA inactivation, resulting in a loss of actin stress fibers and focal adhesions and disassembly of the matrix network [52]. The involvement of microRNA-29 (miR-29) in parallel to β-catenin through the canonical Wnt signaling pathway on extracellular matrix expression in the TM was recently described [20]. However, no miRNA-29 was detected in a AH microRNA profile study, although among the abundant amount of miR-486-5p, miR-204, and miR-184, relatively small amounts of miR181 and miR191 were found [19]. MiR181 and miR191 are reported to be activators of WNT/β-catenin signaling components in epithelial–mesenchymal transition [53]. We speculate that the decrease in β-catenin expression in TM cells mediated by EV might involve miRNAs. The exact miRNA content of NPCE-derived EV remains to be defined.

## Conclusion

In the present study, we characterized NPCE derived EV and studied their effects on the Wnt signaling in TM cells. According to our in-vitro results we suggest that EV originated within the NPCE can influence the trabecular meshwork cadherin's via reduction of phospho-GSK3 and β-catenin expression. This pathway is proposed to be part of the drainage system homeostasis.
